# The Lorazepam and Diazepam Protocol for Catatonia Due to General Medical Condition and Substance in Liaison Psychiatry

**DOI:** 10.1371/journal.pone.0170452

**Published:** 2017-01-23

**Authors:** Chin-Chuen Lin, Yi-Yung Hung, Meng-Chang Tsai, Tiao-Lai Huang

**Affiliations:** Department of Psychiatry, Kaohsiung Chang Gung Memorial Hospital and Chang Gung University College of Medicine, Kaohsiung, Taiwan; Charite Universitatsmedizin Berlin, GERMANY

## Abstract

**Objective:**

The lorazepam-diazepam protocol had been proved to rapidly and effectively relieve catatonia in patients with schizophrenia or mood disorder. This study aims to investigate the efficacy of lorazepam-diazepam protocol in catatonia due to general medical conditions (GMC) and substance.

**Method:**

Patients with catatonia that required psychiatric intervention in various settings of a medical center were included. The lorazepam-diazepam protocol had been used to treat the catatonia due to GMC or substance according to *DSM-IV* criteria. The treatment response had been assessed by two psychiatrists.

**Results:**

Eighteen (85.7%) of 21 catatonic patients due to GMC or substance became free of catatonia after the lorazepam-diazepam protocol. Five (23.8%) of the 21 patients had passed away with various causes of death and wide range of time periods after catatonia.

**Conclusion:**

Our results showed that the lorazepam-diazepam protocol could rapidly and effectively relieve catatonia due to GMC and substance.

## Introduction

Catatonia is a unique neuropsychiatric syndrome of altered consciousness and characteristic psychomotor findings. Catatonia had been associated with schizophrenia, mood disorders, general medical conditions (GMC) [1, 2], substance withdrawal [3–6], and illicit substances [7]. Catatonic patients with known history of mental disorders often receive prompt consultation from psychiatrist, but catatonia due to GMC and substance use is often managed by nonspecialist health professionals. GMC and substance constituted 20–40% of causes of catatonia [8, 9]. Therefore, the timely management of catatonia by non-psychiatrist is important, because delayed treatment can prolong recovery and lead to serious complications such as fetal pulmonary embolism and deep venous thrombosis [10–14].

Previously, we had described a protocol utilizing intramuscular injection (IMI) of lorazepam and intravenous dripping (IVD) of diazepam to effectively and rapidly relieve catatonia in patients with schizophrenia and mood disorders [15–18]. In this treatment protocol, following the initial injection of 4mg of lorazepam, the remaining benzodiazepine (BZD) treatment dosage was converted to diazepam IVD:

1. Lorazepam 1 ampule (2 mg/mL per ampule) IMI.
2. Repeat lorazepam 1 ampule IMI within 2 hours if the first dose failed to achieve complete catatonia relief.
3. Diazepam IVD (10 mg/500ml infused in normal saline at a rate of 1.25mg/hr) continuously until catatonia was completely relieved.

This protocol also succeeded in relieving catatonia associated with GMC in several case reports, such as multiple sclerosis [19], renal insufficiency [20], and uremia [21]. However, no systematic review was performed on this protocol's efficacy in catatonia due to GMC or substance.

In this study, we aim to identify patients with catatonia associated with GMC or substance use who were treated with this lorazepam-diazepam protocol. The mortality of some of these patients are also reviewed.

## Materials and Methods

From July, 1998 to July, 2014, patients with catatonia that required psychiatric consultation in emergency department (ER), intensive care unit (ICU), internal medicine ward, and surgical ward of Kaohsiung Chang Gung Memorial Hospital were recorded in a list maintained by the psychiatric staff. Some of these cases had been previously published [19, 21–23]. Medical records of catatonic patients who were treated with the lorazepam-diazepam protocol were reviewed for their catatonic symptoms and signs, treatments, treatment response, underlying etiologies, and causes of mortality. The institutional review board (IRB) of Chang Gung Memorial Hospital approved the study design of retrospective medical charts review in which no consent was required (IRB 99-2930B). Written informed consents were obtained from surviving patients who agreed to a follow-up interview, which was also approved by IRB (IRB 103-1888B).

Catatonia was diagnosed using *the Diagnostic and Statistical Manual of Mental Disorders*, *Fourth Edition (DSM-IV)*. Specifically, diagnosis was based on the patient meeting at least two of the following five criteria: motor immobility (including waxy flexibility) or stupor, excessive motor activity, extreme negativism or mutism, peculiarities of voluntary movement, and echolalia or echopraxia. Two psychiatrists confirmed the catatonic features. Treatment response was defined as the absence of observed catatonic features according to the *DSM-IV* criteria. Partial response was defined as that catatonic symptoms only improved partially. Resistance to treatment is defined as persistence of catatonic symptoms and signs. The underlying medical etiologies were determined by the internists or surgeons that consulted us. None of the patients had past psychiatric illness. The results are presented as mean ± standard deviation.

## Results

Twenty-one patients diagnosed with catatonia due to GMC (18 patients, 85.7%) or substance-induced catatonia (3 patients, 14.3%) and treated with the lorazepam-diazepam protocol were identified (S1 Table). There were 11 males and 10 females. Their age at the time of catatonia ranged from 21 to 60 years, with a mean of 38.0 ± 10.9 years. Eighteen patients (85.7%) responded favorably with the lorazepam-diazepam protocol. Among them, fourteen (66.7%) became catatonia free after 2mg of lorazepam IMI. One patient required two injections to become catatonia free. Three patients required diazepam IVD after the initial 4mg of lorazepam IMI. Among these three, two patients' catatonia resolved within a day, but one required more than a day. Three patients (14.3%) did not respond well. One patient received two injections of lorazepam but only had her catatonic symptoms partially relieved. Diazepam IVD was not prescribed due to her clinical condition. The other two patients showed virtually no improvement of their catatonic symptoms despite using the protocol for 24 hrs and 7 days, respectively. No obvious side effect such as respiratory suppression was observed among patients receiving the protocol.

The two patients with alcohol withdrawal (cases 18 and 19) were both evaluated and treated in the ER. Both had alcohol dependence for more than ten years, and tried to quit by stopping all alcohol consumption for a few days. They presented with irritability and restlessness initially, and became catatonic one to two days before being sent to ER. Basic lab workup revealed nothing extraordinary, so psychiatry was consulted. Lorazepam-diazepam protocol was utilized to manage their catatonia. After relief of catatonia, one patient (case 18) left ER against medical advice, but was sent back three days later due to vivid auditory hallucination of voices commenting. Alcohol withdrawal delirium was impressed and was managed with BZD, antipsychotic, and thiamine. The other patient (case 19) was admitted after relief of catatonia, but he became agitated and restless along with autonomic instability. Delirium tremens was impressed, and he was managed with BZD, antipsychotic, and thiamine.

Five (27.8%) of the 18 catatonic patients due to GMC had passed away. The three patients with substance related catatonia were well till the day of follow-up. Their data are listed in S2 Table. The causes of mortality were documented in the medical records. Among them, three (60%) showed response to catatonia treatment and two (40%) showed no improvement. One of the two patients with poor treatment response passed away without leaving the hospital, while the other one passed away one month after discharge due to progression of metastatic cancer.

## Discussion

The most important finding of the study is that the lorazepam-diazepam protocol remained effective in relieving catatonia due to GMC or substance. Of the 21 catatonic cases identified, 18 (85.7%) became free of catatonia after the benzodiazepine treatment. The response rate were slightly lower than our previously reported cases of catatonia associated with schizophrenia and mood disorder. The lorazepam-diazepam protocol had a response rate of 60 to 85% within first two hours and 85 to 100% within one day in schizophrenia [17, 18]. In catatonia associated with mood disorders, response rates of 57.1% to 66.7% within the first two hours and 100% within a day were achieved [15, 16]. It is worth noting that 14 out of 18 patients who responded with the protocol became free of catatonia after the initial 2mg of lorazepam injection. Only five out of the 21 patients were prescribed of diazepam IVD, and two of them showed no response to the treatment at all. Nevertheless, this lorazepam-diazepam protocol appeared promising in relieving catatonia due to GMC or substance effectively and rapidly.

Benzodiazepines (BZD) [24, 25] and electroconvulsive therapy (ECT) [26] have been chosen treatments of catatonia, whatever the etiology [27]. BZD should be the first-line treatment for catatonia, then ECT if BZD fails to work [9]. High doses of lorazepam (6–16 mg/day) for 3–5 days relieved catatonia in two thirds of patients, and 2–5 sessions of ECT relieved remaining catatonic signs in the event of BZD failure [25, 28]. Lorazepam dosages up to 30 mg/d might be required [29]. The lorazepam-diazepam protocol, after the initial 2–4 mg of lorazepam IMI, replaces the remaining dosage of lorazepam with diazepam IVD. An advantage of this protocol is its simple administration. While psychiatrists continue to regularly examine patients, nursing staff only need to prepare a bag of 500 c.c. normal saline containing 10mg of diazepam every 8 hours, reducing the clinical workload.

The second finding is that about 30% of the catatonic patients due to GMC had passed away. Their causes of death and survival time after catatonia varied largely, though most responded favorably with the lorazepam-diazepam protocol. There were only few studies investigating long-term prognosis of catatonia. Better outcomes are associated with certain drug prescription for catatonic depression after ECT in a study using telephone interview [30]. A cohort prospective study found that patients with catatonic schizophrenia show a different profile of risk factors and outcomes than those with other subtypes of schizophrenia [31]. In terms of short-term prognosis, the response to high doses of lorazepam at the end of first day could predict the final outcome of a 5-day treatment protocol with lorazepam [25]. Because of multiple confounding factors, a link between their catatonia and their deaths could not be established in this study.

The exact molecular mechanism of catatonia remains unknown. γ-Aminobutyric acid (GABA), dopamine, and *N*-methyl-D-aspartate receptor (NMDAR) dysfunction are some of the most discussed hypothesis regarding the mechanism of catatonia [32–35]. Reduced serum brain-derived neurotrophic factor (BDNF) level had also been reported in patients with catatonic schizophrenia as compared to patients with other subtypes of schizophrenia [36]. Interestingly, after catatonia was relieved by lorazepam, serum BDNF level decreased in patients with schizophrenia [37]. ECT increased plasma but not serum BDNF levels in human subjects [38]. There are also studies regarding cerebral blood flow. Hypoperfusion was observed in the bilateral striatum and the bilateral thalamus of a catatonic patient diagnosed with very-late-onset schizophrenia-like psychosis [39]. ECT increases perfusion in several different brain regions [40, 41], though a similar mechanism might also trigger the emergence of catatonia in a case report [42]. The underlying mechanism of catatonia are still unknown, though it might involve the disturbance of multiple systems.

There are several limitations of this study. The sample size is small, and most likely presents an underestimate of catatonic patients due to GMC and substances. The study design is retrospective and relies on medical charts review. Cause and effects could not be established given the study design is a naturalistic case studies. This analysis focused on catatonic patients treated with the lorazepam-diazepam protocol only. Catatonic patients treated with other treatment modalities, such as ECT, were excluded. The use of intravenous diazepam only relieve catatonia in three out of five patients unresponsive to the initial 4mg of lorazepam. It is possible that intravenous lorazepam would work similarly. Nevertheless, intravenous diazepam was relatively easy to prepare, and our past experiences supported this protocol. Lastly, BZD may cause respiratory suppression, so its use should be closely monitored.

Catatonia presents a unique challenge in ER, ICU, and wards, due to a wide variety of etiologies. In this study, we found that the lorazepam-diazepam protocol could rapidly and effectively relieve catatonia due to GMC and substance, to prevent further complications in already complex clinical situations. The lorazepam-diazepam protocol could effectively and rapidly relieve catatonia, and its simplicity makes it easy to be adopted in various clinical settings.

## Supporting Information

S1 Table Catatonia due to GMC and substance. ARDS: acute respiratory distress syndrome; DM: diabetes mellitus; ESRD: end-stage renal disease; HTN: hypertension; SAH: subarachnoid hemorrhage; SDH: subdural hemorrhage; SLE: systemic lupus erythematus; URI: upper respiratory tract infection; UTI: urinary tract infection.
(DOC)

S2 Table Causes of Mortality. ARDS: acute respiratory distress syndrome; SDH: subdural hemorrhage; UTI: urinary tract infection.
(DOC)
