# Supplementary material for: The Lorazepam and Diazepam Protocol for Catatonia Due to General Medical Condition and Substance in Liaison Psychiatry
Source: PLoS One. 2017 Jan 23;12(1):e0170452. doi: 10.1371/journal.pone.0170452 (PMC5256942; doi:10.1371/journal.pone.0170452)
Supplement: S1 Table — ARDS: acute respiratory distress syndrome; DM: diabetes mellitus; ESRD: end-stage renal disease; HTN: hypertension; SAH: subarachnoid hemorrhage; SDH: subdural hemorrhage; SLE: systemic lupus erythematus; URI: upper respiratory tract infection; UTI: urinary tract infection. (DOC) [file pone.0170452.s001.doc]

**S1 Table. Catatonia due to GMC and substance**

| Cases | Age | Gender | Etiology | Catatonic signs | **Duration (days)** | Treatment | Response | **Associated diagnoses** |  |
| --- | --- | --- | --- | --- | --- | --- | --- | --- | --- |
| 1 | 21 | M | **Viral encephalitis** | Stupor, mutism, negativism, posturing | **14** | Lorazepam IMI x1 | Improved | **Aspiration pneumonia, respiratory failure status post tracheostomy, sepsis, rhabdomyolysis** |  |
| 2 | 27 | F | ARDS | Stupor, mutism, negativism | **2** | Lorazepam IMI x2 + Diazepam IVD <=1 day | Resistant | **Pneumonia** |  |
| 3 | 37 | F | Herpes simplex encephalopathy | Stupor, mutism, negativism | **2** | Lorazepam IMI x2 | Partial | **URI** |  |
| 4 | 34 | M | SAH | Stupor, mutism, negativism | **9** | Lorazepam IMI x2 + Diazepam IVD <=1 day | Improved |  |  |
| 5 | 59 | F | **Diabetic e**ncephalopathy | Stupor, mutism, negativism | **5** | Lorazepam IMI x2 | Improved | **UTI, sepsis, chronic renal failure, DM, HTN** |  |
| 6 | 41 | F | Multiple sclerosis | Stupor, mutism, negativism | **1** | Lorazepam IMI x2 + Diazepam IVD <=1 day | Improved |  |  |
| 7 | 31 | M | Sepsis | Stupor, mutism, catalepsy | **1** | Lorazepam IMI x1 | Improved | **DM** |  |
| 8 | 32 | F | SLE | Stupor, mutism, negativism | **1** | Lorazepam IMI x1 | Improved | **Nephrotic syndrome** |  |
| 9 | 25 | F | Post-partum | Stupor, mutism | **10** | Lorazepam IMI x1 | Improved |  |  |
| 10 | 33 | F | Post-partum | Stupor, mutism | **14** | Lorazepam IMI x1 | Improved |  |  |
| 11 | 38 | F | Post-partum | Stupor, mutism, negativism, stereotypy | **3** | Lorazepam IMI x1 | Improved |  |  |
| 12 | 43 | F | Brain metastasis | Stupor, mutism, negativism, rigidity | **5** | Lorazepam IMI x2 + Diazepam IVD >1 day | Resistant | **Carcinoma of unknown primary origin with lung, bone, liver, and brain metastasis** |  |
| 13 | 39 | M | Wilson's disease | Stupor, mutism | **2** | Lorazepam IMI x1 | Improved |  |  |
| 14 | 31 | M | **Right frontal glioblastoma multiforme** | Stupor, mutism, negativism | **30** | Lorazepam IMI x1 | Improved |  |  |
| 15 | 26 | M | Amphetamine | Mutism, negativism, staring, posturing | **30** | Lorazepam IMI x1 | Improved |  |  |
| 16 | 46 | M | Stroke | Stupor, mutism, posturing | **1** | Lorazepam IMI x1 | Improved | **DM, acute renal failure** |  |
| 17 | 56 | M | ESRD | Stupor, mutism, negativism | **1** | Lorazepam IMI x1 | Improved | **DM, HTN** |  |
| 18 | 32 | M | Alcohol withdrawal | Stupor, mutism, negativism | **1** | Lorazepam IMI x1 | Improved | **Alcohol dependence, alcohol withdrawal delirium** |  |
| 19 | 45 | M | Alcohol withdrawal | Stupor, mutism | **2** | Lorazepam IMI x1 | Improved | **Alcohol dependence, delirium tremens** |  |
| 20 | 42 | M | Uremia | Stupor, mutism | **1** | Lorazepam IMI x2 + Diazepam IVD >1 day | Improved | **Rheumatic heart disease, chronic renal insufficiency** |  |
| 21 | 60 | F | Deep brain stimulation | Stupor, mutism, staring, posturing, rigidity | **1** | Lorazepam IMI x1 | Improved | **Parkinson's disease** |  |

ARDS: acute respiratory distress syndrome; DM: diabetes mellitus; ESRD: end-stage renal disease; HTN: hypertension; SAH: subarachnoid hemorrhage; SDH: subdural hemorrhage; SLE: systemic lupus erythematus; URI: upper respiratory tract infection; UTI: urinary tract infection
