# Supplementary material for: The Lorazepam and Diazepam Protocol for Catatonia Due to General Medical Condition and Substance in Liaison Psychiatry
Source: PLoS One. 2017 Jan 23;12(1):e0170452. doi: 10.1371/journal.pone.0170452 (PMC5256942; doi:10.1371/journal.pone.0170452)
Supplement: S2 Table — ARDS: acute respiratory distress syndrome; SDH: subdural hemorrhage; UTI: urinary tract infection. (DOC) [file pone.0170452.s002.doc]

**S2 Table.** Causes of Mortality

| Cases | Age | Gender | Treatment | Response | Type of Catatonia | Diagnosis | Time of Death | | Causes of Mortality | |  |
| --- | --- | --- | --- | --- | --- | --- | --- | --- | --- | --- | --- |
| 1 | 27 | F | Lorazepam and diazepam | Resistant | Retarded | ARDS | | Same hospitalization | | Respiratory infection | |
| 2 | 59 | F | Lorazepam | Response | Retarded | Encephalopathy | | Same hospitalization | | Respiratory failure | |
| 3 | 42 | F | Diazepam | Response | Retarded | Multiple sclerosis | | 6 months later | | Pneumonia and UTI | |
| 4 | 43 | F | Lorazepam and diazepam | Resistant | Retarded | Metastatic cancer | | One month after discharge | | Metastatic cancer | |
| 5 | 31 | M | Lorazepam | Response | Retarded | Brain tumor | | One year later | | Brain tumor | |

ARDS: acute respiratory distress syndrome; SDH: subdural hemorrhage; UTI: urinary tract infection
